# Metformin restores electrophysiology of small conductance calcium-activated potassium channels in the atrium of GK diabetic rats

**DOI:** 10.1186/s12872-018-0805-5

**Published:** 2018-04-10

**Authors:** Xi Fu, Yilong Pan, Qian Cao, Bin Li, Shuo Wang, Hongjiao Du, Na Duan, Xiaodong Li

**Affiliations:** 1 Department of Cardiology, Shengjing Hospital of China Medical University, Shenyang, 110004 People’s Republic of China; 2 Department of Cardiology, The People’s Hospital of Liaoning Province, Shenyang, 110016 People’s Republic of China

**Keywords:** Small conductance calcium-activated potassium channel, SK channel, Metformin, Diabetes, Arrhythmia

## Abstract

**Background:**

Small conductance calcium-activated potassium channels (SK channels) play a critical role in action potential repolarization in cardiomyocytes. Recently, the potential anti-arrhythmic effect of metformin in diabetic patients has been recognized, yet the underlying mechanism remains elusive.

**Methods:**

Diabetic Goto-Kakizaki (GK) rats were untreated or treated with metformin (300 mg/kg/day) for 12 weeks, and age-matched Wistar rats were used as control (*n* = 6 per group). Electrocardiography, Hematoxylin-eosin staining and Masson’s trichome staining were performed to assess cardiac function, histology and fibrosis. The expression levels of the SK channels in the myocardium were determined by real-time PCR and Western blotting. The electrophysiology of the SK channels in the cardiomyocytes isolated from the three groups of rats was examined by patch clamp assay, with specific blockade of the SK channels with apamin.

**Results:**

Metformin treatment significantly reduced cardiac fibrosis and alleviated arrhythmia in the diabetic rats. In the atrial myocytes from control, GK and metformin-treated GK rats, the expression of KCa2.2 (SK2 channel) was down-regulated and the expression of KCa2.3 (SK3 channel) was up-regulated in the atrium of GK rats as compared with that of control rats, and metformin reversed diabetes-induced alterations in atrial SK channel expression. Moreover, patch clamp assay revealed that the SK current was markedly reduced and the action potential duration was prolonged in GK atrial myocytes, and the SK channel function was partially restored in the atrial myocytes from metformin-treated GK rats.

**Conclusions:**

Our data suggests an involvement of the SK channels in the development of arrhythmia under diabetic conditions, and supports a potential beneficial effect of metformin on atrial electrophysiology.

**Electronic supplementary material:**

The online version of this article (10.1186/s12872-018-0805-5) contains supplementary material, which is available to authorized users.

## Background

According to International Diabetes Federation, the global prevalence of diabetes was 415 million in 2015 and the number is estimated to be 642 million by 2040 [1]. Type 2 diabetes mellitus (T2DM) accounts for 90-95% of all diagnosed cases of diabetes, and it also confers an approximately twofold-increased risk of cardiovascular diseases [2, 3]. In addition to the well-documented coronary artery disease and related cardiac events, cardiac electrical disturbance are recognized as critical cardiovascular complications of T2DM and may lead to tachycardia, fibrillation, cardiomyopathy, and even death [4–6]. However, the mechanisms underlying the relation between T2DM and arrhythmia is not fully understood.

Small conductance calcium-activated potassium channels (SK channels) are important players in cardiac action potential repolarization [7]. The SK channels consists of three isoforms, namely, KCa2.1, KCa2.2 and KCa2.3, which are encoded by *KCNN1*, *KCNN2* and *KCNN3*, respectively. All the three SK isoforms are detected in rat atrial and ventricular myocytes with higher abundance of SK1 and SK2 in the atria than that in the ventricles [8]. A genome-wide study has identified common genetic variants of *KCNN3* that were associated with lone atrial fibrillation [9]. In addition, a selective SK blocker has been shown to significantly prolong atrial refractoriness and reduce atrial fibrillation duration in a canine model of atrial fibrillation [10]. These findings imply an important role of the SK channels in the maintenance of atrial electrophysiology. However, whether the SK channels are involved in T2DM-associated arrhythmia remains to be elucidated.

Metformin is currently considered one of the first-line treatments for T2DM [11]. Recently, the anti-arrhythmic effect of metformin has been gradually recognized. In a 13-year dynamic cohort study of 645,710 patients with T2DM, metformin treatment was associated with a significantly reduced incidence of new-onset atrial fibrillation [12]. It remains unknown whether metformin exerts a direct corrective effect on the electrical activity in the diabetic atrial myocytes. Hence, the current study aims to explore the mechanism underlying the anti-arrhythmic effect of metformin in a T2DM rat model with a special focus on the SK channels.

## Methods

### Animals

Twelve male Goto-Kakizaki (GK) T2DM model rats (10 weeks old, 249.83 ± 4.68 g) were allowed to access to standard chow and water ad libitum and maintained in a controlled environment (22 ± 2 °C, relative humidity of 55 ± 5% and 12,12-h light-dark cycle). The rats’ body weights and blood glucose levels (determined by an Accu-Chek glucometer, Roche) were measured three times a week. Age-matched, male, non-diabetic Wistar rats (249.50 ± 4.48 g) were used as the control (Con). After two-week adaptation, fasting blood glucose and blood insulin levels were measured and an intraperitoneal glucose tolerance test was performed [13] to conform the onset of diabetes in the GK rats. GK rats in the metformin-treated group (Met) (*n* = 6) received intragastric administration of metformin (300 mg/kg/day) since 12 weeks of age for three months, while an equal volume of citric acid buffer was intragastrically administrated daily to the rats in the GK and Con groups (*n* = 6 each group). This high dose of metformin was chosen because that long-term treatment with high-dose metformin could alleviate vascular dysfunction and rescue SK channel-mediated vasodilation in diabetic rats [14, 15]. At the end of three-month metformin treatment, electrocardiogram (ECG) was performed on all rats to monitor the cardiac electrical activity.

### Hematoxylin-eosin and Masson^’^s trichome staining

After the rats were euthanized by i.p. injection with 100 mg/kg pentobarbital sodium, the atrium were excised along with the outline of the atrial appendages, fixed in 4% paraformaldehyde and sectioned. Hematoxylin–eosin and Masson’s staining were performed as previously described [16]. The images of stained atrial sections were captured under a light microscope, and the proportion of fibrotic areas was determined using the NIS-Elements F3.0 software (Nikon, Tokyo, Japan).

### Western blot

To assess the expression levels of SK channels in the atrium, western blotting was conducted according to a previously described method [17]. The primary antibodies used here included anti-KCa2.1 (1:400; Alomone, Israel), anti-KCa2.2 (1:200; Millipore, USA), anti-KCa2.3 (1:500; Abcam, UK), and anti-GAPDH (1,2000; Santa Cruz, USA). GAPDH served as the loading control. ImageJ software (NIH, Bethesda, MD) was used to analyze the intensity of the target bands.

### Real-time PCR

Total RNAs in rat atrial tissues were extracted using an RNeasy kit (Qiagen, CA), and reversely transcribed into cDNA using a PrimeScript RT Reagent Kit with gDNA Eraser (Takara) according to the manufacturer’s instructions. The sequences of the sense (5’-3’) and antisense (5’-3’) primers are as follows: rKCNN1: CCTTCCTGTCCATCGGCTAC, GCGTTTTTAACCCGCTTGGT; rKCNN2: ACTAGCAACTTCCTTGGAGCA, GCAACCTGCACCCATTATTCC; rKCNN3: CACCTTCCCCAAAGCCAACA, CGATCACAAAGAGCTGTACTTCC; rGAPDH: GGCAAGTTCAACGGCACAGT, TGGTGAAGACGCCAGTAGACTC.

### Isolation of rat atrial myocytes and patch-clamp assay

Rat atrial myocytes was isolated as previously described using the Langendorff apparatus [18]. The velocity of heart retrograde perfusion was 1 ml/min. SK currents were apamin sensitive. SK currents = baseline K^+^ current before apamin - K^+^ current after 100 pM apamin (a specific SK channel blocker). According to a standard method [19], whole-cell K^+^ currents in rat atrial myocytes were detected using an EPC10 amplifier, wherein all signals were acquired at 5 kHz and analyzed by Patchmaster software (version 2.72, HEKA Electronics, Lambrecht/Pfalz, Germany).

Quiescent, calcium-tolerant, spindle-shaped cells with clear cross striation were used for action potential recordings at 35 °C. Action potential was elicited as described previously [19]. At the moment of 50 and 90% repolarization, the action potential duration (APD) was detected and recorded as APD_50_ and APD_90_, respectively. In addition, resting potential (RP), action potential amplitude (APA), APD_50_ and APD_90_ were recorded before and after exposure to 100 pM apamin for 15 min. The action potential recordings without apamin exposure showed the normal state, and the results after apamin exposure demonstrated the condition of SK channel blockade.

### Statistical analysis

The data are expressed as mean ± SEM. Control and GK groups were compared using *t* test. For multiple comparisons, one-way analysis of variance (ANOVA) followed by Fisher’s least significant difference test was used when the data conformed Gaussian distribution and homogeneity of variance, and nonparametric Kruskal-Wallis test with Dunn’s multiple comparisons was used otherwise. Statistical analyses were performed using the SPSS software (version 19.0, Chicago, IL) and GraphPad Prism (version 6.0, La Jolla, CA).

## Results

### Metformin alleviated structural remodeling and arrhythmia in the atrium of GK rat

**Fig. 1:**
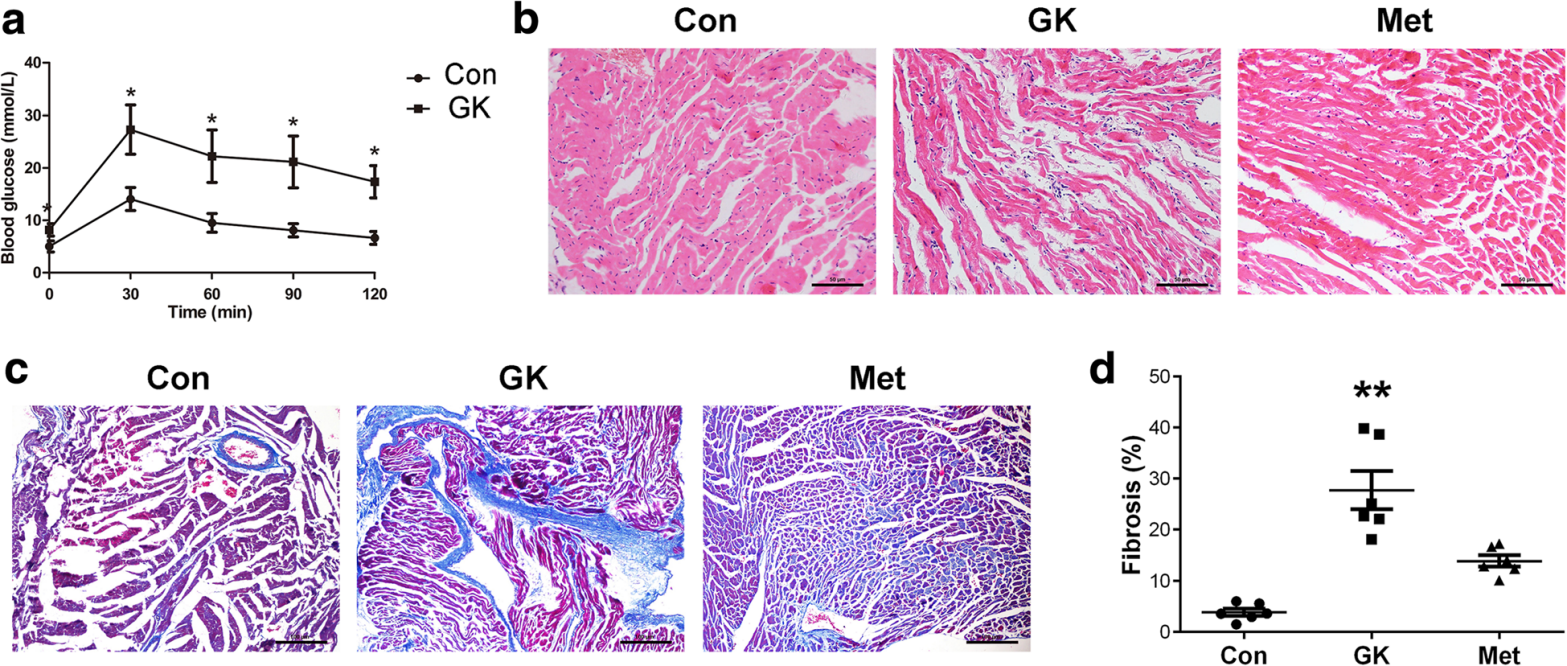
Metformin alleviates histologic changes and fibrosis in GK rat atrium. **a** Blood glucose levels during intraperitoneal glucose tolerance test in control and GK rats. **b** Hematoxylin–eosin staining of rat atrium (scale bar = 50 μm). **c** Masson’s staining of fibrotic tissues in the atrium (scale bar: 100 μm). The collagen fibers were stained blue. **d** Quantification of fibrotic areas as a percentage of total area (*n* = 6 per group). Normal distributed data were represented as mean ± SEM and non-normal distributed data were represented as median with interquartile range; * represents *P* < 0.05 vs Con; **represents *P* < 0.01 vs Con

GK rats, as a T2DM model, displayed hyperglycemia (Table 1) and glucose intolerance (Fig. 1a; Additional file 1: Table S1) by 12 weeks of age, confirming of the diabetic state at the beginning of metformin treatment. After 12-week treatment, the anti-diabetic metformin significantly increased fasting blood glucose and in GK rats, but it also decreased rat body weight (Table 2).

**Table 1:** Diabetic conditions of 12-week old GK rats before metformin treatment

	Con	GK
Weight (g)	249.50 ± 4.48	249.83 ± 4.68
FBG (mM)	4.38 ± 0.24	12.37 ± 0.23**
FINS (mIU/l)	20.46 ± 1.11	27.62 ± 2.53*

*FBG* fasting blood glucose, *FIN* fasting insulin, *n* = 12 per group

*P < 0.05 vs Con, **P < 0.01 vs Con

**Table 2:** Rat body weight and fasting blood glucose level at the end of 12-week metformin treatment

	Con	GK	Met
Weight (g)	519.33 ± 9.64	461.92 ± 4.53*	420.25 ± 5.28*^#^
FBG (mM)	4.31 ± 0.03	13.77 ± 0.16*	9.45 ± 0.05

*FBG* fasting blood glucose, *n* = 6 per group

*P < 0.01 vs Con; ^#^*P* < 0.01 vs GK

**Fig. 2:**
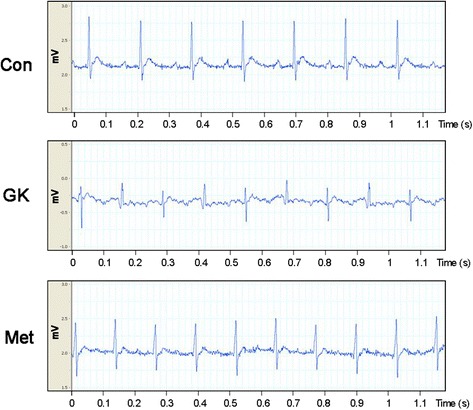
ECG of control, GK and metformin-treated GK rats. GK rats displayed accelerated heart rate, junctional rhythm, separation of *P* and QRS waves, irregular *P* waves and partial blockade of electrical conduction, whereas metformin-treated GK rats showed sinus rhythm and relatively regular *P* waves

Hematoxylin–eosin staining showed that the atrial myocytes in GK rats were disordered and distorted with unevenly stained cytoplasm and irregular nuclei. By contrast, GK rats with metformin treatment showed a less degree of atrial myofilament irregularity (Fig. 1b). Moreover, Masson’s staining revealed that the GK rats developed interstitial fibrosis, and metformin attenuated atrial fibrosis in GK rats (Fig. 1c and d, *P* < 0.01; Additional file 1: Table S2). In the meanwhile, ECG of GK rats showed accelerated heart rate, junctional rhythm, separation of *P* and QRS waves, irregular *P* waves, and partial blockade of electrical conduction (Fig. 2). In contrast, ECG of metformin-treated GK rats showed sinus rhythm and relatively regular *P* waves (Fig. 2). These results demonstrated a protective effect of metformin on the structure and electrophysiology of the atrium under diabetic conditions.

### Metformin modulated the expression of SK channels in the atrium of GK rat

**Fig. 3:**
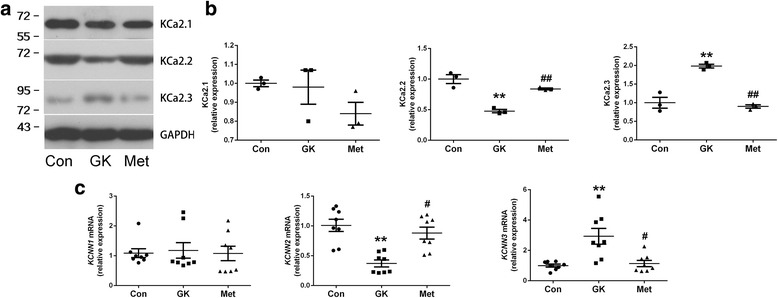
Expression of KCa2.1, KCa2.2 and KCa2.3 in the atrial tissue of control rats and GK rats with or without metformin treatment. **a** Representative Western blots and **b** relative levels of KCa2.1, KCa2.2 and KCa2.3 in the atrial tissues of three groups of rats (*n* = 3 per group). **c** Relative levels of *KCNN1*, *KCNN2* and *KCNN3* mRNAs in the atrial tissues of three groups of rats (*n* = 8 per group). Non-normal distributed data were represented as median with interquartile range; ** represents *P* < 0.01 vs Con; ^#^ represents *P* < 0.05 vs GK; ^# #^ represents *P* < 0.01 vs GK

To investigate the mechanisms underlying diabetic arrhythmia and the anti-arrhythmic effect of metformin, the expressions of the SK channels in the atria of GK rats with and without metformin treatment were examined. Western blot results showed that the level of KCa2.2 in GK atrium was approximately half of that in control atrium and the level of KCa2.3 in GK atrium was 1.98 ± 0.5 fold of that in control atrium (Fig. 3a and b; Additional file 1: Table S3). The expression of KCa2.1 was unchanged in GK atrium compared with control atrium. After 12 weeks of metformin treatment, the expression of KCa2.2 was markedly up-regulated while the expression of KCa2.3 was dramatically down-regulated in the atrium as compared to the untreated GK group (*P* < 0.01).

Next, the mRNA expression levels of the SK channels were measured by real-time PCR. Consistent with the protein levels, the expression of *KCNN2* was down-regulated and the expression of *KCNN3* was up-regulated in the diabetic atrium as compared with control atrium (Fig. 3c, *P* < 0.01; Additional file 1: Table S4). Moreover, metformin treatment significantly increased *KCNN2* expression and decreased *KCNN3* expression in the atrium of GK rats (*P* < 0.05). These results indicated that the expression pattern of the SK channels in rat atrial myocytes was altered under diabetic conditions and metformin abolished diabetes-induced changes in the expression of atrial SK channels.

### SK current decline in diabetic atrial myocytes was reversed by metformin

**Fig. 4:**
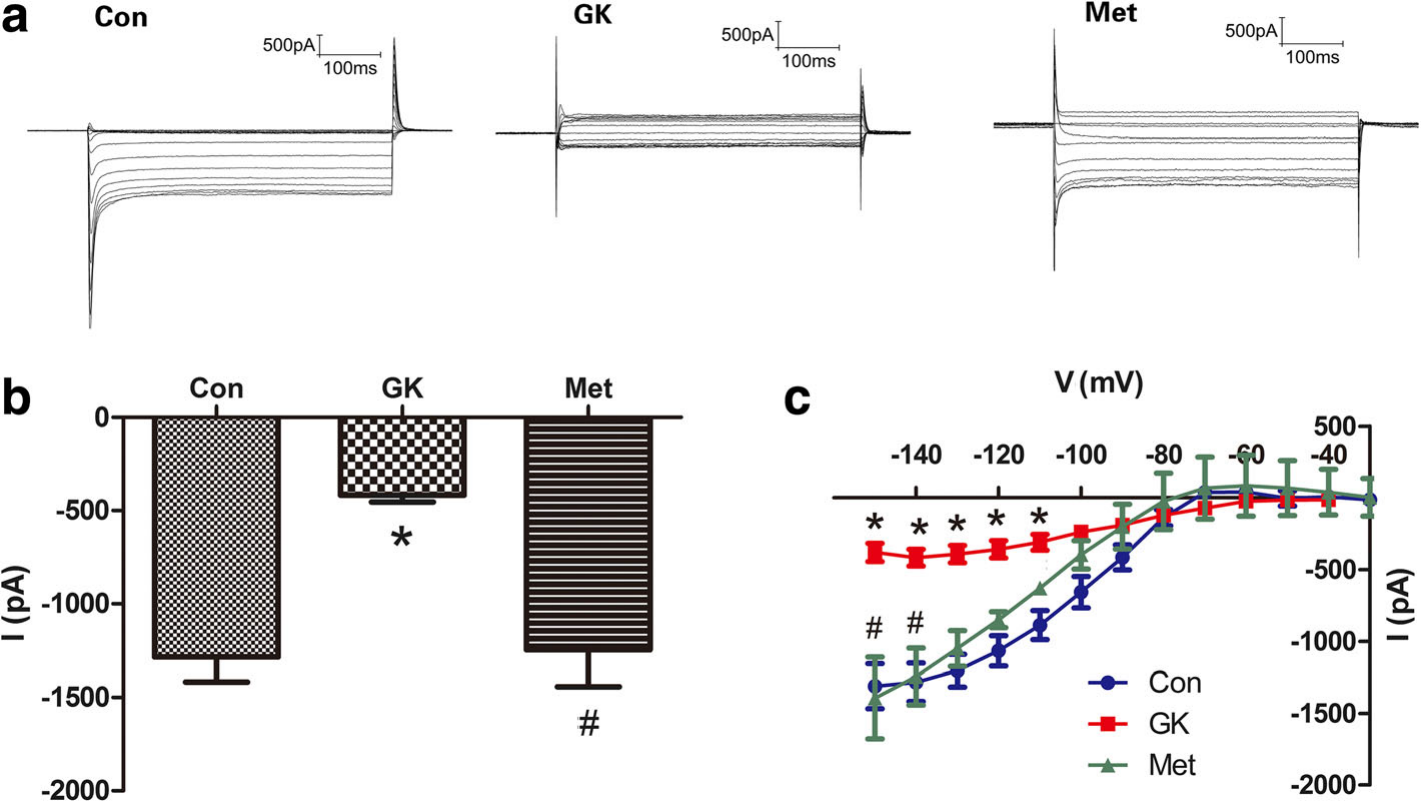
SK current recordings in atrial myocytes from control, GK and metformin-treated GK rats. **a** Representative tracings of SK currents. **b** Mean SK current intensity at − 140 mV. **c** Current-voltage relation of SK currents at voltages ranging from − 150 to − 20 mV. Results are mean ± SEM (*n* = 11-12 cells from three rats in each group); * represents *P* < 0.05 vs Con; ^#^ represents *P* < 0.05 vs GK

The electrophysiological changes in the isolated atrial myocytes of GK rats were assessed by patch clamp. Fig. 4a shows the representative tracings of SK currents in the atrial myocytes from three groups of rats. Compared with control atrial myocytes, atrial myocytes from GK rats displayed a significantly reduced intensity of SK current (*P* < 0.001). By contrast, the SK current was markedly increased in the atrial myocytes from metformin-treated GK rats as compared with the untreated ones (Fig. 4b; Additional file 1: Table S5). In addition, the current-voltage relationship of SK currents was distorted in diabetic atrial myocytes, and it was corrected in the atrial myocytes of GK rats with metformin treatment (Fig. 4c; Additional file 1: Table S6).

### Metformin minimized diabetes-induced lengthening of APD in atrial myocytes

**Fig. 5:**
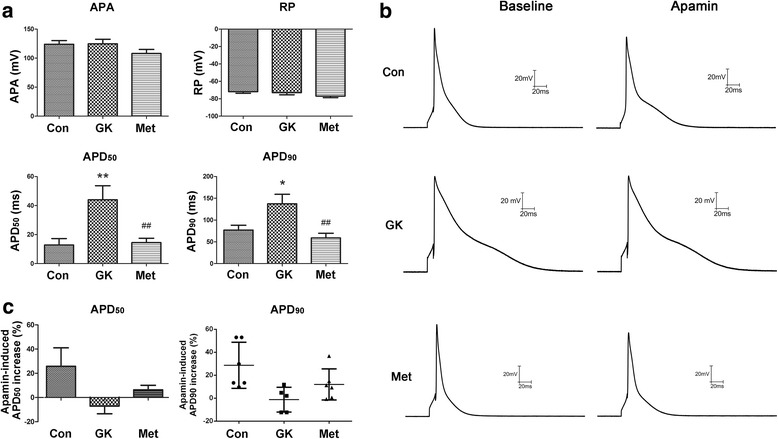
Action potential recordings in atrial myocytes from control, GK and metformin-treated GK rats. **a** Representative action potential parameters (APA, RP, APD_50_ and APD_90_) in freshly isolated atrial myocytes before exposure to apamin. **b** Action potential before and after exposure to apamin (100 pM for 15 min). **c** Changes in APD_50_ and APD_90_ after exposure to apamin. Non-normal distributed data were represented as median with interquartile range (*n* = 11-12 cells from three rats in each group); * represents *P* < 0.05 vs Con; ** represents *P* < 0.01 vs Con; ^# #^ represents *P* < 0.01 vs GK

Next, we measured action potential in the isolated atrial myocytes from three groups of rats. Before exposure to apamin, RP and APA were similar among the three groups; APD_50_ and APD_90_ were significantly prolonged in the GK group (*P* < 0.01), and such lengthening of APD was markedly reduced after metformin treatment (Fig. 5a; Additional file 1: Table S7). Upon exposure to apamin, a specific SK channel blocker, APD_50_ and APD_90_ increased by 25.75 and 26.44%, respectively, in the Con group, and by 6.40 and 11.99%, respectively, in the Met group. However, SK channel blockade had no significant effect on APD in the GK group (Fig. 5b and c; Additional file 1: Table S8). These data implied that the function of the SK channels was impaired in the atrial myocytes of diabetic GK rats, and that the function of atrial SK channels was partially restored after metformin treatment.

## Discussion

Our study demonstrates for the first time that the expression and current intensity of SK channels are altered in the atrial myocytes of diabetic GK rats, which leads to the prolongation of APD. Of course, the changes in APD may result from remodeling of multiple ion channels involving transient outward current and calcium current. By specifically blocking the SK channels with apamin, we found that the atrial myocytes from GK rats did not respond to apamin-induced APD prolongation, indicating that the function of the SK channels was impaired in the atrial myocytes of this T2DM model rat. Consistent with our findings, down-regulation of atrial SK channels in type 1 diabetic mice is associated with a decreased current intensity [20]. In our study, KCa2.2 is the only down-regulated SK channel in diabetic atrium, and it is likely to be responsible for the reduced SK current and prolonged APD in atrial myocytes under diabetic conditions. On the other hand, by taking advantage of a *KCNN3* transgenic mouse model, Zhang et al. demonstrate that overexpression of KCa2.3 results in significant shortening of APD in atrial myocytes and increased susceptibility to atrial fibrillation [21]. Thus, elevated expression of KCa2.3 is a risk factor of atrial fibrillation. Here, the expression of KCa2.3 was significantly increased in the atrium of GK rats, suggesting that it may play a role in diabetes-induced arrhythmia.

The relation between ion channel activity and cardiac electrical property is complicated. Clinical data indicate an increased risk of atrial fibrillation in patients with diabetes [6]. In this study, KCa2.2 was down-regulated while KCa2.3 was up-regulated in the diabetic atrium, implying the potential involvement of the SK channel alteration in the development of arrhythmia or atrial fibrillation. It is reported that down-regulation of KCa2.1 and KCa2.2 is detected along the progression of atrial fibrillation in human [22]. For these differential expression patterns of SK channels, we assume that the expression of SK channels changes with the development of arrhythmia, and the expression pattern depends on the stage of the disease. In addition, blockade of SK channels in various cardiac arrhythmia models has been shown to be both anti-arrhythmic [23] and pro-arrhythmic [7, 24], and both shortening and lengthening of action potential will lead to arrhythmia. Our results showed down-regulation of KCa2.2 and up-regulation of KCa2.3 were associated with prolonged APD in atrial myocytes and arrhythmia in diabetic rats, yet the relationship between each single SK channel and diabetic-associated arrhythmia need to be addressed in the future. Furthermore, our data suggests that diabetic conditions affected SK channel expression at the mRNA level. Therefore, it is important to identify the upstream transcription factors that regulate the expression of SK channels and microRNAs that interfere with the stability of SK channel transcripts [25, 26], and these factors may become potential targets for the intervention of the pathologic changes (such as atrial arrhythmia) in T2DM.

Metabolic stress, inflammation and oxidative stress may affect cardiac functionality and contribute to arrhythmia, and alterations of ion channel activity are implicated in these pathological processes [27]. In the patients affected by outflow tract premature ventricular contractions, a higher recurrence rate after catheter ablation was noticed in those with metabolic syndrome [28]. It is mostly likely that alterations of ion channels in the cardiomyocytes affect the long-term post-ablation prognosis. Here we showed that myocardial expression of SK channels was altered under diabetic conditions. Therefore, in addition to the treatment of arrhythmia in T2DM patients, such as cardiac resynchronization [29, 30], correction of ion channel expression and activity should be considered for the maintenance of cardiac functionality and prevention of recurrence.

In the present study, the anti-arrhythmic effect of metformin was analyzed. Metformin treatment alleviated diabetes-induced atrial remodeling, fibrosis and arrhythmia in GK rats. Moreover, metformin treatment reversed the changes in the expression of SK channels and preserved SK channel function in the atrial myocytes of diabetic rats. Our findings support the beneficial effect of metformin on cardiac electrophysiology under diabetic conditions. Therefore, this anti-diabetic agent not only stabilizes blood glucose in patients with T2DM, but may also provide protection to the heart and prevent diabetic arrhythmia.

## Conclusions

Our study shows that altered expression and function of SK channels altered in atrial myocytes is associated with arrhythmia in a rat model of T2DM. Moreover, metformin treatment may alleviate diabetic arrhythmia by maintaining the expression and function of atrial SK channels.

## Additional file

Additional file 1: **Table S1.** Raw data of blood glucose level. **Table S2.** Raw data of fibrotic area. **Table S3.** Raw data of western blotting detection of the expressions of KCa2.1, KCa2.2 and KCa2.3. **Table S4.** Raw data of real-time PCR detection of the expressions of *KCNN1*, *KCNN2*, and *KCNN3.* **Table S5.** Raw data of SK current intensities at − 140 mV. **Table S6.** Raw data of current-voltage relation of SK currents at voltages ranging from − 150 to − 20 mV. **Table S7.** Raw data of action potential parameters. **Table S8.** Raw data of action potential increase before and after exposure to apamin. (DOC 178 kb)

## Abbreviations

APA: Action potential amplitude
APD: Action potential duration
GK: Goto-Kakizaki
Met: Metformin
RP: Resting potential
SK channel: Small conductance calcium-activated potassium channel
T2DM: Type 2 diabetes mellitus
