# Supplementary material for: Metformin restores electrophysiology of small conductance calcium-activated potassium channels in the atrium of GK diabetic rats
Source: BMC Cardiovasc Disord. 2018 Apr 10;18:63. doi: 10.1186/s12872-018-0805-5 (PMC5894224; doi:10.1186/s12872-018-0805-5)
Supplement: Supplementary file 1 — Table S1. Raw data of blood glucose level. Table S2. Raw data of fibrotic area. Table S3. Raw data of western blotting detection of the expressions of KCa2.1, KCa2.2 and KCa2.3. Table S4. Raw data of real-time PCR detection of the expressions of KCNN1, KCNN2, and KCNN3. Table S5. Raw data of SK current intensities at − 140 mV. Table S6. Raw data of current-voltage relation of SK currents at voltages ranging from − 150 to − 20 mV. Table S7. Raw data of action potential parameters. Table S8. Raw data of action potential increase before and after exposure to apamin. (DOC 178 kb) [file 12872_2018_805_MOESM1_ESM.doc]

**Table S1 Raw data of blood glucose level**

| Group | Time (min) | Blood glucose level (mmol/L) | | | | | | | | | | | |
| --- | --- | --- | --- | --- | --- | --- | --- | --- | --- | --- | --- | --- | --- |
|  |  | Rep1 | Rep2 | Rep3 | Rep4 | Rep5 | Rep6 | Rep7 | Rep8 | Rep9 | Rep10 | Rep11 | Rep12 |
| Con | 0 | 5.9 | 5.2 | 3.6 | 6.2 | 3.7 | 3.9 | 6.1 | 3.6 | 5.5 | 6.4 | 5.1 | 4.9 |
|  | 30 | 16.4 | 17.2 | 15.3 | 10.6 | 15.7 | 11.8 | 12.9 | 16.7 | 12.8 | 11.4 | 14.1 | 13.8 |
|  | 60 | 8.8 | 12.5 | 7.9 | 8.9 | 8.6 | 7.8 | 11.6 | 12.8 | 9.4 | 7.8 | 9.4 | 8.7 |
|  | 90 | 9.8 | 6.1 | 6.5 | 8.9 | 9.2 | 7.2 | 8.1 | 9.4 | 9.2 | 6.5 | 7.9 | 8.3 |
|  | 120 | 7.4 | 4.3 | 6.9 | 8.2 | 6.2 | 5.8 | 5.2 | 7.3 | 8.4 | 5.9 | 6.5 | 7.5 |
| GK | 0 | 8.2 | 10.7 | 8.8 | 7.3 | 8.4 | 9.5 | 7.6 | 6.5 | 6.8 | 8.6 | 7.5 | 8.1 |
|  | 30 | 29.6 | 34.2 | 32.2 | 17.9 | 25.3 | 30.7 | 28.6 | 27.4 | 19.8 | 27.9 | 27.5 | 26.3 |
|  | 60 | 25.0 | 30.4 | 27.8 | 13.7 | 19.4 | 26.1 | 25.7 | 20.6 | 14.7 | 21.6 | 21.3 | 20.4 |
|  | 90 | 24.3 | 29.8 | 15.4 | 18.6 | 26.1 | 24.2 | 26.5 | 17.5 | 13.9 | 17.4 | 20.4 | 19.6 |
|  | 120 | 17.8 | 20.7 | 21.4 | 12.1 | 16.7 | 21.5 | 18.4 | 15.4 | 12.8 | 15.6 | 18.7 | 17.1 |

**Table S2 Raw data of fibrotic area**

| Group | Fibrotic area (%) | | | | | |
| --- | --- | --- | --- | --- | --- | --- |
|  | Rep1 | Rep2 | Rep3 | Rep4 | Rep5 | Rep6 |
| Con | 6.01 | 3.66 | 2.98 | 5.61 | 3.59 | 1.50 |
| GK | 22.6 | 22.06 | 25.09 | 39.84 | 18.11 | 38.63 |
| Met | 10.16 | 12.91 | 14.06 | 17.23 | 12.45 | 16.66 |

**Table S3 Raw data of western blotting detection of the expressions of KCa2.1, KCa2.2, and KCa2.3**

| Protein | Group | Relative expression level | | |
| --- | --- | --- | --- | --- |
|  |  | Rep1 | Rep2 | Rep3 |
| KCa2.1 | Con | 1.03 | 0.97 | 1.00 |
|  | GK | 1.07 | 0.80 | 1.07 |
|  | Met | 0.77 | 0.79 | 0.96 |
| KCa2.2 | Con | 1.04 | 0.86 | 1.10 |
|  | GK | 0.46 | 0.44 | 0.53 |
|  | Met | 0.87 | 0.83 | 0.82 |
| KCa2.3 | Con | 1.28 | 0.94 | 0.78 |
|  | GK | 2.07 | 1.89 | 1.99 |
|  | Met | 0.81 | 0.92 | 0.97 |

**Table S4 Raw data of real-time PCR detection of the expressions of *KCNN1*, *KCNN2*, and *KCNN3***

| Protein | Group | Relative expression level | | |  |  |  |  |  |
| --- | --- | --- | --- | --- | --- | --- | --- | --- | --- |
|  |  | Rep1 | Rep2 | Rep3 | Rep4 | Rep5 | Rep6 | Rep7 | Rep8 |
| *KCNN1* | Con | 1.02 | 1.12 | 0.96 | 0.90 | 2.08 | 0.77 | 0.87 | 0.99 |
|  | GK | 2.46 | 0.93 | 0.77 | 0.81 | 2.26 | 0.78 | 0.74 | 0.67 |
|  | Met | 1.35 | 1.85 | 0.53 | 0.48 | 1.27 | 2.18 | 0.47 | 0.48 |
| *KCNN2* | Con | 0.95 | 0.61 | 1.33 | 1.11 | 0.97 | 0.59 | 1.24 | 1.29 |
|  | GK | 0.46 | 0.57 | 0.21 | 0.23 | 0.45 | 0.60 | 0.23 | 0.22 |
|  | Met | 1.19 | 1.07 | 0.51 | 0.75 | 1.10 | 1.16 | 0.53 | 0.73 |
| *KCNN3* | Con | 1.27 | 1.28 | 0.99 | 1.06 | 1.23 | 0.51 | 0.90 | 0.75 |
|  | GK | 4.07 | 2.56 | 3.86 | 2.33 | 2.51 | 5.55 | 1.40 | 1.16 |
|  | Met | 1.54 | 2.26 | 0.84 | 0.70 | 1.04 | 1.44 | 0.65 | 0.63 |

**Table S5 Raw data of SK current intensities at -140 mV**

| Group | SK current intensity (pA)(×10^12^) | | | | | | | | | | | |
| --- | --- | --- | --- | --- | --- | --- | --- | --- | --- | --- | --- | --- |
|  | Rep1 | Rep2 | Rep3 | Rep4 | Rep5 | Rep6 | Rep7 | Rep8 | Rep9 | Rep10 | Rep11 | Rep12 |
| Con | -907 | -1465 | -1528 | -998 | -1519 | -1428 | -892 | -1487 | -1889 | -956 | -965 | -1384 |
| GK | -538.99 | -312.52 | -498.33 | -536.99 | -301.52 | -444.33 | -423.76 | -321.23 | -439.26 | -476.78 | -417.73 | -302.26 |
| Met | -1650 | -832 | -2046 | -863 | -817 | -1022 | -823 | -1665 | -1265 | -807 | -1467 | -1692 |

**Table S6 Raw data of current-voltage relation of SK currents at voltages ranging from -150 to -20 mV**

| Group | Rep No. | SK currents (pA)(×10^12^) | | | | | | | | | | | | | |
| --- | --- | --- | --- | --- | --- | --- | --- | --- | --- | --- | --- | --- | --- | --- | --- |
|  |  | -150 | -140 | -130 | -120 | -110 | -100 | -90 | -80 | -70 | -60 | -50 | -40 | -30 | -20 |
| Con | Rep1 | -905 | -907 | -861 | -782 | -680 | -536 | -369 | -369 | 18.6 | 39.9 | 13.3 | -24.3 | -50.7 | -54.6 |
|  | Rep2 | -1142 | -1465 | -1260 | -1170 | -1030 | -848 | -564 | -216 | -0.09 | -79.4 | -250 | -8.6 | -62.23 | -34 |
|  | Rep3 | -904 | -1529 | -842 | -743 | -650 | -516 | -324 | -387 | 18.2 | 39.2 | 12.9 | -29.3 | -18.76 | -52.73 |
|  | Rep4 | -1598 | -998 | -1470 | -1340 | -1150 | -897 | -582 | -234 | 6.33 | 41.9 | 26 | -7.9 | -19.2 | -27 |
|  | Rep5 | -1117 | -1519 | -1259 | -1179 | -1054 | -898 | -563 | -201 | -0.13 | -81.3 | -265 | -8.9 | 82 | -62.36 |
|  | Rep6 | -898 | -1428 | -835 | -782 | -680 | -536 | -369 | -140 | 18.6 | 39.9 | 13.3 | -24.3 | -50.7 | -54.6 |
|  | Rep7 | -1503 | -1487 | -1462 | -1340 | -1150 | -897 | -582 | -234 | 6.33 | 41.9 | 26 | -7.09 | -19.2 | -27 |
|  | Rep8 | -1839 | -892 | -1319 | -986 | -636 | -234 | -32.4 | 130 | 178 | 158 | 148 | 104 | 74 | 72.7 |
|  | Rep9 | -1772 | -1891 | -1498 | -1399 | -1156 | -913 | -626 | -242 | 6.42 | 40.9 | 28 | 119 | -48.45 | 86 |
|  | Rep10 | -1342 | -954 | -1288 | -756 | -654 | -214 | -369 | -137 | 19.43 | 41.2 | 15.8 | -5.6 | -19.37 | -23.7 |
|  | Rep11 | -1821 | -965 | -1503 | -1324 | -1180 | -522 | -587 | -245 | 4.74 | 42.3 | 29 | -6.5 | -11.21 | -21.2 |
|  | Rep12 | -895 | -1384 | -846 | -996 | -628 | -896 | -32.7 | 147 | 179 | 156 | 156 | -3.8 | -21.16 | -23.7 |
| GK | Rep1 | -476 | -538.99 | -464.23 | -427.25 | -340.03 | -250.95 | -178.23 | -101.43 | -53.9 | 9.87 | 33.11 | 35.57 | NA | NA |
|  | Rep2 | -260 | -312.52 | -270.99 | -239.82 | -203.03 | -156.28 | -134.21 | -97.84 | -66.84 | -63.25 | -67.71 | -63.83 | NA | NA |
|  | Rep3 | -405 | -498.33 | -441.78 | -412.62 | -386.07 | -309.46 | -257.9 | -167.81 | -95.07 | -21.25 | -9.17 | -12.2 | NA | NA |
|  | Rep4 | -513 | -547.99 | -464.23 | -427.43 | -337.54 | -209.43 | -178.43 | -100.54 | -49.34 | 10.64 | 39.34 | 39.23 | NA | NA |
|  | Rep5 | -487 | -287.52 | -271.99 | -211.65 | -201.58 | -137.46 | -134.65 | -97.52 | -67.54 | -69.24 | -72.57 | -67.54 | NA | NA |
|  | Rep6 | -268 | -444.33 | -439.78 | -434.76 | -411.32 | -264.76 | -257.75 | -178.65 | -95.26 | -19.46 | -11.42 | -14.57 | NA | NA |
|  | Rep7 | -216 | -423.76 | -496.23 | -411.54 | -397.65 | -331.31 | -178.76 | -101.63 | -51.35 | 11.39 | 36.67 | 36.81 | NA | NA |
|  | Rep8 | -418 | -321.23 | -231.91 | -227.43 | -175.21 | -171.65 | -112.54 | -89.26 | -64.46 | -63.64 | -69.79 | -61.42 | NA | NA |
|  | Rep9 | -403 | -439.26 | -451.74 | -445.34 | -401.42 | -251.42 | -275.43 | -167.91 | -101.83 | -21.13 | -9.83 | -9.93 | NA | NA |
|  | Rep10 | -493 | -476.78 | -496.31 | -412.67 | -316.65 | -262.93 | -178.53 | -97.52 | -51.25 | 10.27 | 41.25 | 38.46 | NA | NA |
|  | Rep11 | -226 | -417.73 | -257.87 | -211.21 | -179.98 | -161.64 | -107.97 | -91.47 | -64.88 | -62.48 | -68.57 | -69.77 | NA | NA |
|  | Rep12 | -414 | -302.26 | -421.01 | -457.01 | -364.91 | -337.31 | -297.42 | -183.49 | -101.68 | -21.39 | -13.66 | -11.95 | NA | NA |
| Met | Rep1 | -1325 | -1650 | -1292 | -1035 | -703 | -596 | -537 | -432 | -428 | 802 | -364 | -333 | -308 | 67.8 |
|  | Rep2 | -1830 | -832 | -1300 | -986 | -636 | -234 | -32.4 | 130 | 178 | 158 | 148 | 104 | 84 | 72.7 |
|  | Rep3 | -968 | -2046 | -842 | -749 | -655 | -563 | -469 | -389 | -345 | -376 | -287 | -250 | -240 | -196 |
|  | Rep4 | -2300 | -863 | -1400 | -985 | -524 | -93.1 | 321 | 664 | 832 | 846 | 751 | 643 | 471 | 354 |
|  | Rep5 | -905 | -817 | -861 | -782 | -680 | -536 | -369 | -140 | 18.6 | 39.9 | 13.3 | -24.3 | -50.7 | -54.6 |
|  | Rep6 | -968 | -1022 | -842 | -749 | -655 | -563 | -469 | -389 | -345 | -312 | -306 | -250 | -240 | -213 |
|  | Rep7 | -1358 | -823 | -792 | -723 | -601 | -91 | 301 | 387 | 803 | -323 | 697 | 254 | 96 | -68.9 |
|  | Rep8 | -1478 | -1665 | -1258 | -986 | -667 | -269 | -39.4 | 156 | 149 | 36 | 148 | 249 | 94 | 72.7 |
|  | Rep9 | -937 | -1265 | -811 | -737 | -634 | -572 | -504 | -389 | -389 | -354 | -397 | -250 | -79 | -206 |
|  | Rep10 | -2897 | -807 | -1531 | -976 | -512 | -98 | 297 | 684 | 723 | 811 | 692 | 632 | 555 | 375 |
|  | Rep11 | -887 | -1456 | -837 | -765 | -665 | -559 | -412 | -189 | 9.6 | 41.9 | 11.3 | -24.3 | -50.7 | -54.6 |
|  | Rep12 | -879 | -1692 | -832 | -732 | -635 | -593 | -497 | -392 | -393 | -362 | -299 | -287 | -297 | -196 |

**Table S7 Raw data of action potential parameters**

| Parameter | Group | Rep No. | | | | | | | | | | | |
| --- | --- | --- | --- | --- | --- | --- | --- | --- | --- | --- | --- | --- | --- |
|  |  | Rep1 | Rep2 | Rep3 | Rep4 | Rep5 | Rep6 | Rep7 | Rep8 | Rep9 | Rep10 | Rep11 | Rep12 |
| APA (mV) | Con | 112.87 | 141.06 | 126.49 | 114.91 | 109.87 | 115.76 | 124.76 | 109.23 | 127.65 | 128.87 | 149.24 | 123.87 |
|  | GK | 145.37 | 98.77 | 120.85 | 139.67 | 118.47 | 142.63 | 95.73 | 126.86 | 138.81 | 121.73 | 139.78 | 109.98 |
|  | Met | 81.10 | 128.68 | 113.81 | 98.66 | 103.42 | 129.99 | 76.78 | 126.72 | 112.67 | 98.86 | 105.73 | 108.78 |
| RP (mV) | Con | -68.81 | -72.95 | -72.45 | -69.82 | -69.37 | -71.83 | -76.87 | -69.67 | -63.78 | -72.83 | -71.84 | -72.73 |
|  | GK | -75.39 | -64.91 | -76.83 | -69.14 | -78.30 | -77.94 | -62.87 | -75.87 | -70.79 | -73.87 | -75.83 | -68.83 |
|  | Met | -71.39 | -79.23 | -72.81 | -83.09 | -76.75 | -78.05 | -71.47 | -78.78 | -73.87 | -84.87 | -79.92 | -71.34 |
| APD_50_ (ms) | Con | 4.30 | 9.42 | 33.97 | 5.44 | 3.79 | 5.78 | 6.31 | 6.32 | 28.87 | 5.37 | 39.93 | 4.39 |
|  | GK | 21.03 | 51.47 | 30.80 | 79.26 | 39.23 | 18.42 | 54.84 | 32.42 | 79.78 | 39.87 | 51.42 | 19.32 |
|  | Met | 23.22 | 5.25 | 11.97 | 13.49 | 9.73 | 26.17 | 20.78 | 5.63 | 12.47 | 8.76 | 21.47 | 19.78 |
| APD_90_ (ms) | Con | 73.79 | 68.71 | 123.33 | 41.67 | 71.48 | 68.78 | 129.63 | 69.63 | 76.74 | 129.84 | 38.71 | 31.48 |
|  | GK | 72.00 | 147.20 | 106.52 | 197.33 | 163.38 | 69.87 | 151.78 | 121.67 | 199.84 | 68.74 | 178.67 | 167.78 |
|  | Met | 109.48 | 32.59 | 53.15 | 47.59 | 49.60 | 61.52 | 111.23 | 26.47 | 51.74 | 49.78 | 47.78 | 65.47 |

**Table S8 Raw data of action potential increase before and after exposure to apamin**

| Parameter | Group | Rep No. | | | | | |  |  |  |  |  |  |
| --- | --- | --- | --- | --- | --- | --- | --- | --- | --- | --- | --- | --- | --- |
|  |  | Rep1 | Rep2 | Rep3 | Rep4 | Rep5 | Rep6 | Rep7 | Rep8 | Rep9 | Rep10 | Rep11 | Rep12 |
| APD_50_ (%) | Con | 9.35 | 2.45 | 21.07 | 74.13 | 7.38 | 4.43 | 20.32 | 74.43 | 4.45 | 75.42 | 3.43 | 4.42 |
|  | GK | -22.41 | 16.02 | -11.6 | -5.12 | -4.55 | -24.42 | 18.32 | -20.42 | -6.45 | -4.64 | -26.42 | -3.73 |
|  | Met | 20.15 | 5.84 | -5.82 | -0.31 | 11.21 | 7.3 | 22.83 | 5.42 | -5.32 | -0.25 | 12.78 | 7.54 |
| APD_90_ (%) | Con | 52.96 | 13.35 | 9.61 | 29.85 | 52.96 | 12.35 | 56.87 | 11.98 | 7.54 | 26.78 | 53.98 | 14.34 |
|  | GK | -12.39 | 4.69 | 11.82 | 2.01 | -12.37 | -11.34 | 7.43 | 12.42 | 5.56 | -9.42 | -10.42 | -11.73 |
|  | Met | 11.28 | 36.67 | -0.8 | 0.6 | 9.26 | 14.89 | 10.78 | 38.76 | -0.9 | -0.7 | 9.24 | 13.78 |
